# Full Recovery From Cocaine-Induced Toxic Leukoencephalopathy: Emphasizing the Role of Neuroinflammation and Brain Edema

**DOI:** 10.1177/2324709619868266

**Published:** 2019-08-13

**Authors:** Edward C. Mader, Alexander B. Ramos, Roberto A. Cruz, Lionel A. Branch

**Affiliations:** 1 Louisiana State University Health Sciences Center, New Orleans, LA, USA

**Keywords:** toxic, leukoencephalopathy, white matter, edema, inflammation, cocaine, MRI, EEG

## Abstract

Toxic leukoencephalopathy (TL) is characterized by white matter disease on magnetic resonance imaging (MRI) and evidence of exposure to a neurotoxic agent. We describe a case of cocaine-induced TL in which extensive white matter disease did not preclude full recovery. A 57-year-old man with substance abuse disorder presented with a 5-day history of strange behavior. On admission, he was alert but had difficulty concentrating, psychomotor retardation, and diffuse hyperreflexia. Brain MRI revealed confluent subcortical white matter hyperintensities with restricted diffusion in some but not in other areas. Electroencephalography (EEG) showed mild diffuse slowing. Blood tests were normal except for mild hyperammonemia. Urine screen was positive for cocaine and benzodiazepine but quantitative analysis was significant only for cocaine. Prednisone 60-mg qd was initiated on day 4, tapered over a 5-day period, and discontinued on day 9. He was discharged after 3 weeks. Cognitive function returned to normal 2 weeks after discharge. Five months later, neurologic exam and EEG were normal and MRI showed near-100% resolution of white matter lesions. TL has been attributed to white matter ischemia/hypoxia resulting in demyelination/axonal injury. The clinical, EEG, and MRI findings and time course of recovery of our patient suggest that cocaine-induced inflammation/edema resulted in TL but not in ischemic/hypoxic injury. While inflammation/edema may have regressed when cocaine was discontinued, we cannot exclude a role for prednisone in protecting the patient from the ischemic/hypoxic sequelae of inflammation/edema. MRI is indispensable for diagnosing TL but EEG may provide additional useful diagnostic and prognostic information.

## Introduction

Toxic leukoencephalopathy (TL) is suspected whenever a person develops neurobehavioral symptoms, and magnetic resonance imaging (MRI) reveals white matter lesions, especially if the lesions are widespread, confluent, and symmetric.^1,2^ Diagnosing TL requires evidence of exposure to an agent with known white matter toxicity. It is also important to exclude other causes of leukoencephalopathy, such as autoimmune, inflammatory, or infectious white matter diseases, posterior reversible encephalopathy syndrome, chronic microvascular changes, and inherited disorders of myelin metabolism.^3,4^ A variety of chemicals can potentially cause TL, including substances of abuse (eg, heroin, opiates, amphetamines, cocaine, and ethanol), antineoplastic drugs (eg, methotrexate, cisplatin, and fluorouracil), immune modulators (eg, cyclosporine and interferon), and environmental toxins (eg, carbon monoxide, toluene, arsenic, and carbon tetrachloride).^1-3^ The MRI findings can be highly characteristic with some of these neurotoxins such that one can suspect the offending agent based on MRI results. For example, carbon monoxide neurotoxicity with bilateral lobar white matter lesions will also cause damage to the anterior globus pallidus with or without the involvement of the thalamus, caudate, putamen, and cerebellum (for a pictorial review see Refs 3 and 4). The clinical spectrum of TL is broad, with symptoms varying from inattention, forgetfulness, and personality change, to delirium and coma.^1,2^ Clinical outcome is also variable.^5^ Although death has been reported many times, survival is still the rule. Survivors may achieve full recovery after a short or long period of convalescence or continue to experience variable degrees of neurological deficits.^1,2,5^ The earlier the offending agent is identified and discontinued, the better the prognosis.

Cocaine-induced TL has been reported much less frequently than TL caused by other drugs of abuse, notably heroine and other opioids.^6-18^ There is apparently no significant difference in the risk of TL between chronic cocaine abusers and first-time users as well as between individuals who inhale cocaine and those who smoke it in the form of crack.^19^ Some reported cases of cocaine-induced TL resulted in death or in major functional impairment.^6,11,13,14,18^ The overall mortality rate of cocaine-induced TL is about 23%.^19^ Some survivors who went into remission experienced one or more relapses with repeat or continuous exposure to cocaine.^8,10^ At the opposite end of the spectrum are patients with cocaine-induced TL who experienced full recovery or who developed minimal residual deficits.^7-9,12,16,17^ Some received corticosteroid (unspecified), dexamethasone, or methylprednisolone with plasmapheresis or with intravenous immunoglobulins.^8,10,12,17^ However, there are also patients who were treated with corticosteroid (unspecified), methylprednisolone, cyclophosphamide, and/or plasma exchange with poor outcome.^11,13-15^ Interestingly, cocaine-induced TL was the final diagnosis in a few patients who were initially thought to have neuroleptic malignant syndrome, schizophrenia-induced catatonia, Susac’s syndrome, or Balo’s concentric sclerosis.^7,11,19,20^ White matter lesions have also been detected in the MRI of asymptomatic cocaine users.^19^

The variability in symptoms, MRI findings, disease severity, and clinical outcomes in the reported cases of cocaine-induced TL may be due to the fact that cocaine is not always the ingredient causing TL. For example, the adulterant levamisole or the pasta base of cocaine have been implicated in the pathogenesis of TL.^9-11,13,14,16^ Nevertheless, even if cocaine were the actual cause of TL in all of the reported cases, the clinicopathological profile would probably still be heterogeneous because cocaine-induced brain injury is mediated via different pathophysiological mechanisms. Some patients with cocaine-induced TL presented with mild symptoms despite evidence of severe disease on MRI.^12,15^ We describe a case of cocaine-induced TL with complete clinical recovery despite MRI evidence of extensive white matter disease. The time course of clinical recovery, electroencephalographic (EEG) normalization, and resolution of MRI lesions suggest that cocaine-induced neuroinflammation and white matter edema occurred without significant demyelination and axonal injury.

## Case Presentation

A 57-year-old man with substance abuse disorder was brought by his family to the emergency room because of strange behavior. Five days prior to admission, he fell off his bicycle and came home with a scalp laceration on the left side of the head. Since then, his family noticed him acting oddly. He was seen walking back and forth in the house between the front door and the back door. He emptied the ice tray and he poured sugar into a shopping bag for no reason. He could no longer perform habitual tasks, such as cooking. His family described his gait as slow “like a zombie.” It was also unusual for him not to leave the house 5 days in a row. He does not see a primary care physician. According to his family, he smoked tobacco regularly (2 packs/day for 40 years), consumed alcohol rarely, and abused marijuana and cocaine.

The patient did not complain of headache, nausea, dizziness, and so on; he thinks nothing is wrong with him. Blood pressure was 187/104 mm Hg, heart rate 77 beats/min, respiratory rate 16 breaths/min, temperature 36.6°C, and blood oxygen saturation 100%. Criteria for hypertensive emergency or urgency were not met. He was awake and alert with normal thought content and intact ability to answer questions, but his affect was flat, he was indifferent to his condition, and he exhibited psychomotor retardation. On neurological examination, he was oriented to self, place, and immediate situation, but was oblivious of the month, day, and year. His ability to concentrate was mildly impaired and he pauses frequently when speaking; nevertheless, speech and language function were intact. Cranial nerve functions and motor strength were all normal. Deep tendon reflexes were brisk (3+) in all extremities. Ankle clonus and Babinski sign were absent. Cerebellar signs were also absent. Head computed tomography showed mild diffuse brain atrophy. Blood counts and chemistry were all normal except for mild hyperammonemia (39 µmol/L).

**Figure 1.**
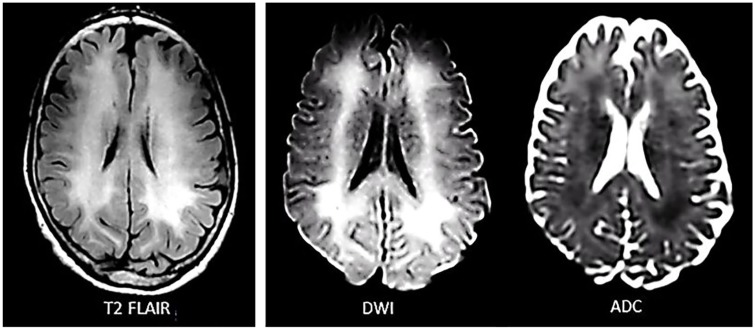
Inpatient brain MRI during the acute stage of cocaine-induced toxic leukoencephalopathy (hospital day 3; 8 days after onset of symptoms) showed widespread and symmetric T2-FLAIR and DWI hyperintensities in the subcortical white matter with low ADC in some areas (restricted diffusion) and high ADC in other areas (T2 shine-through effect). The cerebral cortex was spared and the cerebellum, basal ganglia, thalamus, and brainstem were all normal (the latter structures are not shown).

**Figure 2.**
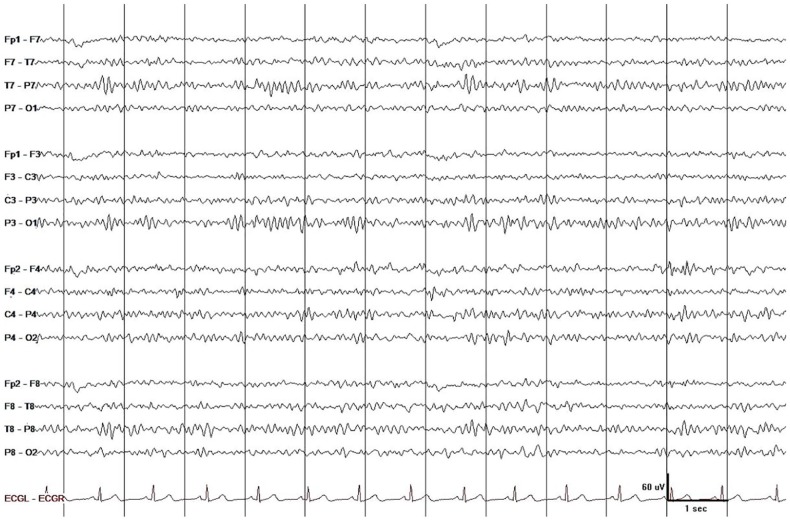
EEG showed mild diffuse slowing with inordinate amounts of arrhythmic asynchronous theta waves mixed with low-voltage beta and alpha activity. This finding is a good match with the mild neuropsychiatric manifestations and preservation of consciousness but not with the widespread white matter abnormality on MRI. The latter if caused by widespread demyelination or axonal injury would result in more prominent slow wave activity. Display parameters: longitudinal bipolar montage (from top to bottom: LT-LP-RP-RT-ECG), digital filter bandpass of 1 to 70 Hz, and 60-Hz notch filter turned on; voltage-time scale is superimposed on the tracing.

He was admitted to the neurology service. Urine was positive for cocaine and benzodiazepine, but only cocaine was found significant on quantitative analysis (levamisole was not tested). Additional studies, including thyroid stimulating hormone; folate; B_12_; HgbA1C; HIV; hepatitis panel; heavy metal screen for lead, arsenic, and mercury; and blood toxicology for ethanol, methanol, salicylates, acetaminophen, and so on, were all negative. Brain MRI revealed diffuse confluent subcortical white matter hyperintensities on T2-FLAIR and diffusion-weighted imaging (DWI) with low-apparent diffusion coefficient (ADC) in some areas (restricted diffusion) and high ADC in other areas (T2 shine-through effect). The cerebral cortex, cerebellum, basal ganglia, thalamus, and brainstem were all normal (Figure1). EEG showed mild diffuse slowing with inordinate amounts of theta waves intermixed with low-voltage beta and alpha activity (Figure 2). Lumbar puncture was considered but not pursued because we were convinced that he has cocaine-induced TL and we could not justify exposing him to the risk of brain herniation. Albeit infrequent, brain herniation may occur with diffuse brain edema (eg, liver failure).^21^

**Figure 3.**
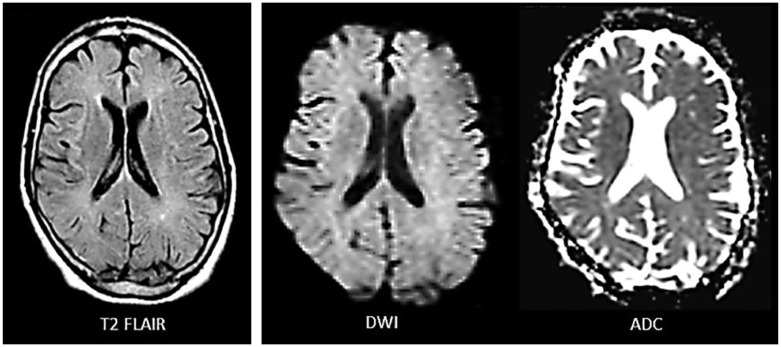
Outpatient brain MRI performed 5 months after the initial inpatient MRI (compare with Figure 1) showed near-100% resolution of subcortical white matter hyperintensity on T2-FLAIR. Diffusion-weighted imaging showed complete resolution of restricted diffusion (normal DWI and ADC).

**Figure 4.**
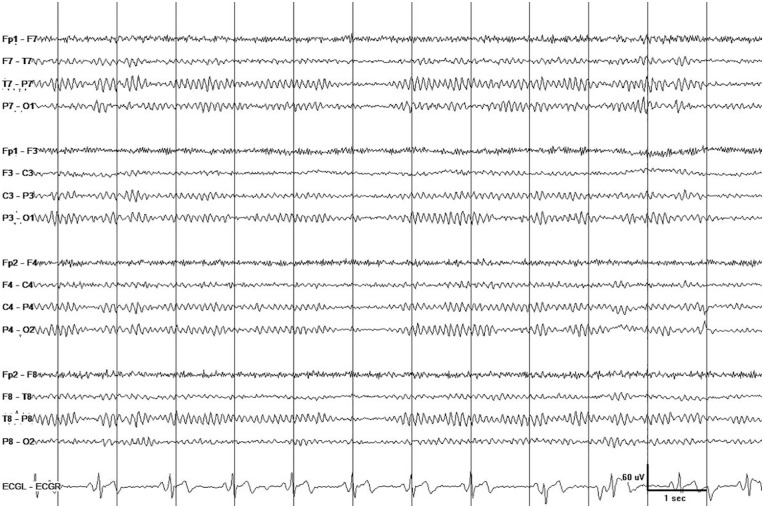
Outpatient EEG recorded 5 months after recording the initial inpatient EEG (compare with Figure 2) showed normal alpha rhythm posteriorly and normal beta activity anteriorly during wakefulness. Display parameters: longitudinal bipolar montage (from top to bottom: LT-LP-RP-RT-ECG), digital filter bandpass of 1 to 70 Hz, and 60-Hz notch filter turned on; voltage-time scale is superimposed over the tracing.

Immunosuppressive therapy in cocaine-induced TL has been associated with a favorable^8,10,12,17^ or unfavorable^11,13-15^ outcome (see “Introduction”). Notwithstanding this uncertainty, we chose to treat the patient with a 5-day course of prednisone because of the extent of his disease. Prednisone 60-mg qd was administered on hospital day 4, tapered to 20 mg qd over 5 days, and stopped on hospital day 9. Nutritional therapy and assistance with activities of daily living resulted in gradual normalization of mental status and behavior. After 3 weeks in acute care, the patient was discharged to a rehabilitation facility. He missed his appointments and was seen again in clinic after 5 months. His family pointed out that his cognitive function returned to normal 2 weeks after he was discharged from acute care and at that point he started engaging again in the activities of daily living. During clinic visit, he reported (and his family agreed) that he no longer takes cocaine and other drugs of abuse. Neurological examination and cognitive evaluation did not detect any abnormalities. Follow-up brain MRI showed complete absence of diffusion restriction and near-100% resolution of white matter hyperintensity (Figure 3). Follow-up EEG showed normal alpha rhythm posteriorly and normal beta activity anteriorly during wakefulness (Figure 4).

## Discussion

Cocaine alters the brain’s physiology and the person’s behavior by binding to dopamine transporter and inhibiting the reuptake of dopamine and other neurotransmitters.^22^ The neurotoxic effects of cocaine are, however, more complex and involve a variety of mechanisms, including dopamine-mediated increase in free radicals and oxidative stress, glutamate-mediated excitotoxicity, changes in gene transcription, and induction of neuroinflammation.^23^ In the case presented, chronic cocaine abuse resulted in acute TL with mild symptoms (disorientation to time, difficulty concentrating, and psychomotor retardation), mild EEG changes (scattered theta waves mixed with normal rhythms), and prominent MRI abnormalities (extensive white matter disease with restricted diffusion in some areas but not in others). Complete cognitive and functional recovery was achieved in 5 weeks. EEG normalized and MRI showed near-100% resolution of white matter lesions in 5 months (perhaps over a shorter time period since he missed his follow-up appointments). Whatever pathophysiological mechanism is proposed for cocaine-induced TL in our patient must explain the evolution and time-course of recovery of all clinical and laboratory findings.

Poor outcome in TL has be attributed to ischemic/hypoxic injury of the white matter resulting in demyelination and axonal degeneration.^24,25^ In light of the mild electroclinical abnormalities and rapid clinical recovery (5 weeks), ischemic/hypoxic white matter injury is not a suitable explanation for TL in our patient. The best pathophysiologic explanation is cocaine-induced white matter inflammation and edema that was not severe enough to cause significant ischemic/hypoxic injury of the white matter, thus sparing our patient persistent neurological deficits. Arguably, TL was not complicated by ischemia/hypoxia in our patient because inflammation/edema started to regress the moment access to cocaine was lost during hospitalization. Some authors pointed out the importance of early removal of the offending agent in the prognosis of TL.^1,26^ There is also a possibility that the anti-inflammatory anti-edema effects of prednisone averted the ischemic/hypoxic sequelae of inflammation and edema. Some authors have already suspected a role for steroids, plasmapheresis, or intravenous immunoglobulins in improving the clinical outcome of cocaine-induced TL.^8,10,12,17^

The patient’s brain MRI showed extensive white matter disease (T2-FLAIR and DWI hyperintensity) with restricted diffusion (low ADC) in some areas but not in others (high ADC; Figure 1). Intracellular edema resulting from metabolic failure (eg, ischemia/hypoxia) is known as cytotoxic edema.^27^ The term “cytotoxic” is misleading since it implies that cell swelling inevitably leads to cell death, which is not always true.^28^ Intracellular edema can occur in astrocytes, oligodendrocytes, neurons, and other cells in the brain. In fact, astrocytes swell up faster than neurons.^27^ Intramyelinic edema, which is by and large reversible, is also described as “cytotoxic.”^29^ Extracellular edema due to breakdown in blood-brain barrier (BBB), allowing plasma proteins (mainly albumin) and water to enter the extracellular fluid (ECF) space, is known as vasogenic edema.^30^ Another form of extracellular edema, known as ionic edema, is characterized by the accumulation of water and ions (not proteins) in the ECF space due to increased BBB permeability or due to a rise in fluid pressure within the perivascular space or cerebrospinal fluid compartment.^30,31^ Areas of restricted diffusion in the patient’s white matter could represent astrocytic, axonal, oligodendrocytic, and/or intramyelinic edema. Vasogenic edema could have been the reason for the absence of restricted diffusion in parts of the white matter with hyperintensity. However, extensive vasogenic edema would have increased the patient’s intracranial pressure (ICP) leading to headache, nausea, and vomiting, or depressed sensorium. Since our patient only had mild cognitive deficits and no clear-cut symptoms of increased ICP, ionic edema is a more suitable explanation for the white matter hyperintensities without restricted diffusion. The original classification of cerebral edema—the one that is familiar to clinicians—does not include ionic edema. As a matter of fact, it is only in recent years that the concept of ionic edema has drawn some clinical interest. Both types of brain edema, intracellular and extracellular, can be accounted for by cocaine-induced neuroinflammation.

**Figure 5.**
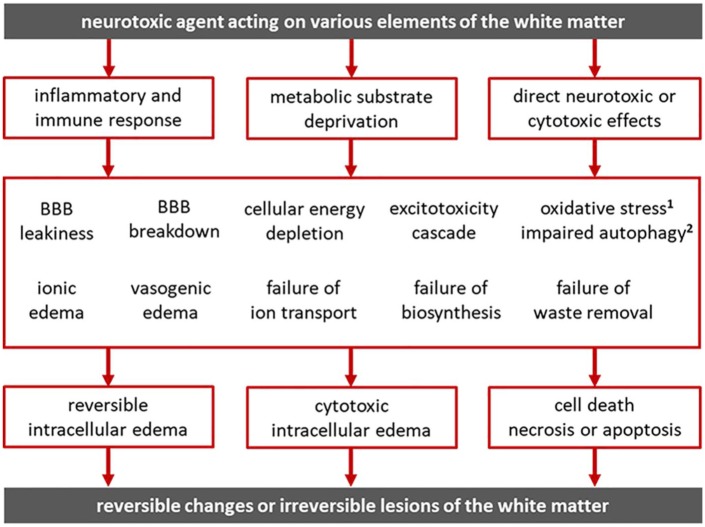
Neurotoxic agents give rise to subcortical white matter lesions by activating inflammatory processes, by depriving cells of essential metabolic substrates (oxygen, glucose, thiamine, etc), or by directly interfering with cellular functions. Neuroinflammation can affect the blood-brain barrier (BBB) directly or through inflammatory mediators and result in ionic edema, vasogenic edema, reversible cell swelling, or cytotoxic edema. Interference with substrate/nutrient delivery to cells, as in the case of ischemia, hypoxia, hypoglycemia, nutrient deficiency, nutrient diffusion impedance from edema, and so on, can result in metabolic supply-demand imbalance, failure of energy metabolism, energy depletion, ion pump failure, and activation of detrimental processes leading to apoptosis or necrosis. These pathophysiological processes interact in complex ways to produce white matter lesions with varying degrees of reversibility. ^1^Antioxidant mechanisms defend the cell against *oxidative stress* from free radicals. ^2^Autophagy protects the cell from *endoplasmic reticulum (ER) stress* due to misfolded proteins.

Neurotoxic agents can produce subcortical white matter lesions by activating inflammatory processes, by impeding substrate delivery and depriving cells of oxygen, glucose, thiamine, and so on, or by directly interfering with cell function (Figure 5). The pathophysiological mechanisms that result in white matter lesions interact in complex ways to produce white matter lesions with varying degrees of reversibility. Cocaine can induce neuroinflammation, impede metabolic substrate delivery via vasoconstriction, and directly interfere with cell physiology.^23^ Microglial cells, astrocytes, and pericytes are activated by cocaine-induced impairment of autophagy leading to neuroinflammation.^32-34^ Autophagy protects cells from the endoplasmic reticulum stress caused by misfolded proteins and other defective products of biosynthesis.^33,34^ By exposing endothelial cells to oxidative stress, cocaine can compromise the integrity of the BBB and lead to brain edema.^35^ Cocaine-induced neuroinflammation and production of inflammatory mediators can also affect the BBB and result in ionic edema, vasogenic edema, reversible cell swelling, or cytotoxic edema. When activated by cocaine, glial cells produce interleukins and cytokines-inducing cell migration to the site of injury.^23,35^ BBB disruption due to cytokine release and endothelial cell dysfunction can facilitate the transmigration of leukocytes and human immunodeficiency viruses from the blood to the brain parenchyma.^36^ Cocaine-induced dopamine signaling can reduce glutamate reuptake by astrocytes and lead to stimulation of glial and immune cells thus amplifying the inflammatory response.^37^ Apparently, the neuroinflammatory, ischemic/hypoxic, and direct neurotoxic effects of cocaine interact in complex ways to produce white matter lesions with varying degrees of reversibility.

The variability in TL disease severity and prognosis depends on the mechanisms of neurotoxicity, the magnitude and duration of exposure, the intensity of cellular response (based on genetics and previous exposures), and the presence of confounding factors, such as other drugs, dehydration, metabolic disturbances, and so on. Various pathophysiological processes that give rise to white matter lesions may coexist, overlap, or interact such that what could have been a reversible injury could become irreversible (Figure 5). For example, vasogenic edema increases the capillary-to-cell distance and the concentration of albumin in the ECF space reducing the rate of effective solute diffusion and interfering with nutrient delivery to the cells.^30,31^ If severe, vasogenic edema can trigger a rise in ICP and result in ischemic injury and permanent sequela.^30,31^ While all the mechanisms shown in Figure 5 may play a role in cocaine-induced TL, neuroinflammation and BBB leakiness resulting in highly reversible extracellular and intracellular edema would be the best explanation for the toxic effects of cocaine in our patient. Full recovery and near-100% resolution of white matter lesions on MRI argue against demyelination and axonal injury. As mentioned earlier, cocaine avoidance and/or steroid therapy early in the stage of illness may have spared our patient the ischemic/hypoxic sequelae of TL.

EEG is often performed in PRES (due to the high probability of seizures) but not in patients with TL.^38^ During the acute stage of TL, the patient’s EEG showed scattered theta waves. Five months later, his EEG was completely normal. Since it took him 5 months to return and get a repeat EEG, it is possible that EEG normalization occurred earlier, for example, around the time his cognition started to normalize. As a rule, EEG is not very useful in the investigation of brain edema because water accumulation in the ECF space has minimal or no effects on the EEG.^39^ Indeed, vasogenic edema will affect the EEG only if there is significant ICP elevation or parenchymal injury.^40^ On the other hand, cytotoxic edema will almost always affect the EEG. These basic principles imply that EEG can be useful in the workup of acute diffuse leukoencephalopathy because it can provide the clinician with some clues regarding the dominant mechanism responsible for the white matter disease. No EEG change or minimal slowing (as in the case presented) would suggest white matter edema, whereas substantial slow wave activity, often with polymorphic morphology, would indicate demyelination/axonal injury.^41^ EEG should be utilized in TL for this reason, as well as for detecting nonconvulsive status epilepticus and for tracking recovery of brain function during the course of TL.

## Conclusion

This case demonstrates that cocaine-induced toxic leukoencephalopathy, even when extensive, does not preclude full functional recovery if the electroclinical profile is favorable, if cocaine is stopped immediately, and (perhaps) if immunotherapy is administered early in the course of the disease. The time course of clinical recovery, EEG normalization, and disappearance of MRI lesions in our patient suggests that the main pathophysiological mechanism underlying toxic leukoencephalopathy was neuroinflammation resulting in extracellular and intracellular white matter edema. It also suggests that the extracellular white matter edema was predominantly “ionic” due to BBB leakiness rather than “vasogenic” due BBB breakdown and that the intracellular white matter edema was predominantly “reversible” glial cell, axonal, and/or intramyelinic edema rather than “irreversible” cytotoxic edema due to ischemic/hypoxic injury. EEG, which is sensitive to white matter disease that disturbs signal transmission along subcortical fiber tracts but barely affected by white matter edema, may provide useful adjunctive diagnostic and prognostic information regarding toxic leukoencephalopathy.
